# Our December Number

**Published:** 1895-12

**Authors:** 


### OUR DECEMBER NUMBER.

We crave the indulgence of our readers this month in presenting to them so many pages devoted to the subject of tuberculosis, but we do this because there is no question of so much importance at this time pressing for solution. It reaches every city, town, village, hamlet, and household the world over. It involves the standing of the veterinary profession at home and abroad. It adds to-day an importance to our calling and its usefulness scarcely conjectured ten years ago. It means much for the future of those who are to obtain a livelihood in veterinary channels. It is new light, it is original research, it is important in results, it is the completion of plans by which we are to render assistance to our people, and of which every veterinarian should be fully acquainted, so that he may join with the people of his town, city, or State in considering methods and plans for dealing with this gravely important subject on a wise, conservative, and economical plan. We believe that after reading these many contributions to this subject that all will join with us in the opinion that they feel mere fully equipped as progressive veterinarians than ever before.

